# Challenges and solutions for transforming health ecosystems in low- and middle-income countries through artificial intelligence

**DOI:** 10.3389/fmed.2022.958097

**Published:** 2022-12-02

**Authors:** Diego M. López, Carolina Rico-Olarte, Bernd Blobel, Carol Hullin

**Affiliations:** ^1^Research Group in Telematics Engineering, Telematics Department, University of Cauca, Popayán, Colombia; ^2^Medical Faculty, University of Regensburg, Regensburg, Germany; ^3^eHealth Competence Center Bavaria, Deggendorf Institute of Technology, Deggendorf, Germany; ^4^First Medical Faculty, Charles University Prague, Prague, Czechia; ^5^Digital Innovation Center of Latin America, Temuco, Chile; ^6^Data Governance Unit, Victoria Legal Aid, Melbourne, VIC, Australia

**Keywords:** artificial intelligence, healthcare systems, low-and-middle income countries, scoping review, implementation challenges

## Abstract

**Background:**

Recent studies demonstrate the potential of Artificial Intelligence to support diagnosis, mortality assessment, and clinical decisions in low-and-middle-income countries (LMICs). However, explicit evidence of strategies to overcome the particular challenges for transformed health systems in these countries does not exist.

**Objective:**

The present study undertakes a review of research on the current status of artificial intelligence (AI) to identify requirements, gaps, challenges, and possible strategies to strengthen the large, complex, and heterogeneous health systems in LMICs.

**Design:**

After introducing the general challenges developing countries face, the methodology of systematic reviews and the meta-analyses extension for scoping reviews (PRISMA-ScR) is introduced according to the preferred reporting items. Scopus and Web of Science databases were used to identify papers published between 2011–2022, from which we selected 151 eligible publications. Moreover, a narrative review was conducted to analyze the evidence in the literature about explicit evidence of strategies to overcome particular AI challenges in LMICs.

**Results:**

The analysis of results was divided into two groups: primary studies, which include experimental studies or case studies using or deploying a specific AI solution (*n* = 129), and secondary studies, including opinion papers, systematic reviews, and papers with strategies or guidelines (*n* = 22). For both study groups, a descriptive statistical analysis was performed describing their technological contribution, data used, health context, and type of health interventions. For the secondary studies group, an in-deep narrative review was performed, identifying a set of 40 challenges gathered in eight different categories: data quality, context awareness; regulation and legal frameworks; education and change resistance; financial resources; methodology; infrastructure and connectivity; and scalability. A total of 89 recommendations (at least one per challenge) were identified.

**Conclusion:**

Research on applying AI and ML to healthcare interventions in LMICs is growing; however, apart from very well-described ML methods and algorithms, there are several challenges to be addressed to scale and mainstream experimental and pilot studies. The main challenges include improving the quality of existing data sources, training and modeling AI solutions based on contextual data; and implementing privacy, security, informed consent, ethical, liability, confidentiality, trust, equity, and accountability policies. Also, robust eHealth environments with trained stakeholders, methodological standards for data creation, research reporting, product certification, sustained investment in data sharing, infrastructures, and connectivity are necessary.

**Systematic review registration:**

[https://rb.gy/frn2rz].

## Introduction

Information and Communication Technologies (ICT), in particular artificial intelligence (AI), is transforming health services, research, and public health in many countries (1). In its WITFOR Vilnius Declaration from 2003 already, the International Federation for Information Processing (IFIP) World Information Technology Forum (WITFOR), supported by the UNESCO, described the challenges and solutions in the context of impacts resulting from information and communication technologies as follows:

• **Bridging** the digital divide between rich and poor in the world; urban and rural societies; men and women; and different generations
• **Ensuring** the freedom of expression enshrined in Article 19 of the universal declaration of human rights and other such instruments
• **Reducing** poverty through the use of education and Information and Communications Technology (ICT)
• **Facilitating** the social integration of excluded segments of societies
• **Respecting** linguistic and cultural diversity
• **Fostering** the creation of public domains with full respect for intellectual property rights
• **Supporting** communities in fighting illiteracy
• **Encouraging** e-governance and e-democracy initiatives
• **Improving** the quality of life through effective health service systems
• **Protecting** the local and global environment for future generations.

In low-and-middle-income countries (LMICs), the use of these technologies can help to close the gaps in healthcare, especially in underserved regions that lack healthcare specialists, as well as improve public health surveillance (2). In addition, the United Nations has estimated that different digital health technologies, including AI, can help countries achieve the Sustainable Development Goals and reach the universal health services coverage goal (3). The WITFOR Vilnius Health Commission highlighted the inclusion of IT strategies in health care to target the major health problems in LMICs, such as HIV/AIDS, TB, Malaria, and mother and child health. LMICs should therefore prioritize Health Information Systems, using multiple sources of aggregated and anonymized data from different related sectors in society, aiming at strengthening health management and primary health care delivery, including a basic hospital structure (4). Integration within and between healthcare establishments requires consistent specification of data sets and terminology. Future health information systems should optimally use Free and Open Source Software, models, and component specifications characterized by scalability and flexibility through a component-based architecture enabling the free combination of relevant services allowing for incremental development; portability separating logical and technological specifications, and a fine-grained architecture to manage complexity. Furthermore, sustainable systems must be based on: training and institutional development enabling local adaptation, maintenance, and use; leadership of health professionals and other domain experts in systems development; and must focus on the local use of information for action (5).

Autonomous systems and artificial intelligence significantly transform health and social care ecosystems (6). This paper especially addresses artificial intelligence for transformed health systems in low- and middle-income countries–frequently still called developing countries.

### Rationale

Artificial intelligence (AI) has permeated all spheres of the development of scientific, social, and cultural knowledge of humanity. One accepted definition of AI is the capability of computers to mimic human cognition, becoming able to learn, reason, understand, adapt, self-regulate and interact with the environment (7, 8). In addition, some experts propose that artificial intelligence manifests itself through appropriately obtaining its goals; second, flexibility to change; third, learning from experience; fourth, making appropriate decisions (9, 10).

Artificial intelligence has changed how we communicate and interact with our environment. It can also improve and strengthen essential areas for the survival of humankind, such as food, transportation, education, and health. In particular, the health sector has experienced growth in AI research. The processes of promoting healthy habits, prevention, diagnosis, and treatment of diseases have been transformed to improve the effectiveness of these processes. Furthermore, the early detection of health threats from the environment or human activity, such as COVID-19, has benefited from AI development and deployment (11, 12).

Formal research differentiating the challenges and comparing the level of AI development between higher-income settings and LMICs was not found. However, the World Health Organization (WHO) highlights the importance of implementing technologies to guarantee universal access to health care and improve the living conditions of communities around all member countries (13). Particularly AI has the potential to improve patient care, diagnoses, and treatment and improve public health efficiency of health systems in high, medium, and low-income countries (14). Moreover, a recommendation on digital health adoption from the Organization for Economic Cooperation and Development (OECD), an international organization created to promote the economic health of members countries which are mainly high-income countries, claim the urgent need to develop policy to regulate ICT use, improve structures, and invest in human and institutional capacity. Those are the core challenges identified in this scoping review.

Low-and-middle-income countries are communities that do not have affordable and accessible healthcare services. Therefore, the potential for AI to help close the gaps in healthcare provision is clear. According to (15), in 2019, around 60% of the world’s population lacked access to even essential healthcare. Also, (16, 17) declared that 8.4 million lives had been lost each year and $1.6 trillion in productivity in LMICs where poor health care quality is provided. Moreover, there are significant challenges surrounding AI implementation for healthcare in LMICs, as recently described by WHO in their guidance on Ethics and Governance of Artificial Intelligence for Health (14). One concrete example is digitizing medical and health records. Such records have the primary input of AI, which is the demographic and clinical data (10, 18). In addition, incorporating the results of the decision-making support systems into the processes in the health facilities reduces the workload of health workers (19, 20). Other critical challenges are ethical and regulatory issues. Special considerations regarding informed consent, security, privacy, trust, liability, confidentiality, equity, and accountability policies must be taken.

However, paradoxically, another significant challenge to effectively implementing AI in LMICs for healthcare comes from the vast and extensive development and deployment of AI in High-Income Countries (HICs). Since data used in the production of AI systems are highly linked to the context of use, implementing such systems in LMICs can result in contextual bias. According to (15), contextual bias means the development of predictive AI models trained with data not reflecting the real context of the use of the algorithms, which can be considered a threat to the promise of AI to foster healthcare democratization of health services in LMICs because the models trained with the wrong data, cannot be used to build decision support tools for primary healthcare practitioners, that way overcoming the shortage of specialized health care professionals. Moreover, the fact that ML models are created in HICs can drive inequality, concentrating wealth, resources, and decision-making power in the hands of a few countries, companies, or citizens (21).

Last but not least, AI-based models must be trained and deployed on a well-developed legal and regulatory framework tailored to the public health systems needs of LMICs. It would allow a careful adoption of these technologies and a positive impact on healthcare systems by addressing the biological and demographic differences of the population. Otherwise, AI could reinforce and exacerbate health and socioeconomic disparities (22). The process of managing AI should not be limited to solving health problems individually; implementing AI should be visualized as an object of transformation in LMICs’ health systems (8).

### Objectives

With the above in mind, this review aims to collect, identify and analyze the gaps and challenges of implementing AI in the healthcare systems of LMICs and provide possible solutions to strengthen health systems and overcome the challenges. There is a set of 40 challenges gathered in eight different domains that affect the stakeholders in the healthcare system.

The paper is organized according to the Preferred Reporting Items for Systematic reviews and Meta-Analyses extension for Scoping Reviews (PRISMA-ScR) Checklist (23).

## Methods

### Protocol and registration

The protocol for this scoping review was drafted according to a pre-defined objective: to identify and analyze gaps, challenges, and possible solutions and strategies to strengthen health systems in LMICs through the proper development of AI. Within the protocol, there were detailed criteria to search and include/exclude sources of evidence and explain the search approach in the selected databases with a proper justification for choices. Once this was clear to reviewers, there was a consensus on extracting the data. The protocol provided the plan for the study selection process, including resolving disagreements between reviewers and the draft charting table for data extraction with accompanying explanations. The protocol was developed following the JBI Reviewer’s Manual (24) and was finally revised on November 3rd, 2021. The protocol can be found in the URL: https://rb.gy/frn2rz.

### Eligibility criteria

For studies to be included in this scoping review, they only needed to describe an AI solution or focus on discussing the gaps and challenges of developing AI for health systems in LMICs. We did not want to discard relevant contributions assessing their methodological robustness. Therefore, any study that could be classified as a research contribution according to the Equator (Enhancing the QUAlity and Transparency Of health Research) Network for which reporting guidelines exist (e.g., Observational studies, Systematic reviews, Diagnostic/prognostic studies, Case reports, Clinical practice guidelines, Qualitative research, Quality improvement studies, Economic evaluations). Peer-reviewed journal papers were included if they were published between 2011 and 2022, written in English or Spanish, and developed an AI application for specific health purposes (communicable diseases, maternal and newborn health, or cancer, among others), especially in LMICs. Studies with datasets were included and classified according to the data origin. Review articles, meta-analyses, and opinion papers were also included because they were considered relevant to the narrative review section. Moreover, the paper was included if any strategy or guideline for properly implementing AI was presented. Articles were excluded if they did not meet the mentioned criteria or were study or review protocols.

### Information sources

Scopus and Web of Science (WoS) databases were used to perform the search to identify potentially relevant documents. Although PubMed supports searching and retrieving biomedical and life sciences literature, Scopus covers all journals in PubMed. The search strategy was drafted considering the review question and objective defined in the protocol. The terms for the search strings were (i) artificial intelligence and its synonyms or contained concepts (e.g., machine learning or data science), and (ii) the concept of low and middle-income countries and their synonyms (low-resource settings). Further refinement of the search strategy was made through team discussion. The studies were searched in the databases on November 8th, 2021. The final search results were exported into Bibliometrix (25), a tool for bibliometric analysis, where duplicates were removed.

Two complementary information sources were consulted for the data extraction process and classification of documents. First, the WHO health topic classification (26) was used to classify the health purpose approach in the included studies. The World Bank Country and Lending Groups Classification (27) was also used to obtain more information on the data and contributions origin since the study’s objective was focused on LMICs.

### Search

Description of the search strings used in Scopus and WoS and their results, are shown in Table 1. The strings were refined through two discussion rounds. Finally, the search was performed without any restrictions on the database (except for the year limitation established as eligibility criteria). Refining the search string allowed us to obtain wider results from Scopus and more restricted figures from WoS, as shown in Table 1.

**TABLE 1:** Information on string search.

ID	String search	Scopus	WoS
1	TITLE-ABS-KEY [(“artificial intelligence” OR “machine learning” OR “deep learning”) AND (lmic OR “low-resource settings” OR “low- and middle-income”)]	285	
	[TS = (artificial intelligence OR machine learning OR deep learning)] AND TS = (lmic OR low-resource settings OR low- and middle-income)		230
2	TITLE-ABS-KEY [(“artificial intelligence” OR AI OR “machine learning” OR “deep learning” OR “data analytics” OR “data science”) AND (lmic OR “low-resource settings” OR “low- and middle-income”)]	**311**	
	TS = (“artificial intelligence” OR AI OR “machine learning” OR “deep learning” OR “data science” OR “data analytics”) AND TS = (lmic OR “low-resource settings” OR “low- and middle-income”)		**161**

### Selection of sources of evidence

The reviewers screened 331 papers after removing duplicates. The screening process consisted of evaluating each publication by title and abstract; if the documents met the eligibility criteria, contributions, author’s affiliations, and datasets used were considered. Finally, the identified relevant studies were classified according to the aim provided in the abstract’s paper.

The eligibility of papers depended on the type of study identified. For this scoping review, sources of evidence were divided into two groups to facilitate the analysis of the results. The first group included experimental or case studies (those describing a specific AI solution); the second group comprised strategy or guidelines papers, opinion papers, or secondary studies like systematic reviews.

After identifying the type of study, a score from 1 to 4 was given to each paper according to the relevance of contributions from the study. Classification criteria for the primary studies group were:

• Score 1: Studies only describing a model-based solution (i.e., machine/deep learning model or natural language processing) or dataset building for a health context not located in LMICs.
• Score 2: Studies describing a data science solution (i.e., machine/deep learning or natural language processing implementations from feature extraction until deployment) or a model-based solution within a device for a health context not located in LMICs.
• Score 3: Studies only describe a model-based solution (i.e., machine/deep learning model or natural language processing) or dataset building for a health context in LMICs.
• Score 4: Studies describing a data science solution (i.e., machine/deep learning implementation from feature extraction until deployment) or a model-based solution with a device for a health context located in LMICs.

Classification criteria for the secondary studies group were:

• Score 1: Papers not describing any challenge (e.g., requirements, gaps, limitations, or barriers) nor proposing any solution (e.g., strategy, framework, initiative, policy, recommendation, or guideline) for AI use or implementation in LMICs.
• Score 2: Papers describing challenges (e.g., requirements, gaps, limitations, or barriers); however, no solution was proposed (e.g., strategy, framework, initiative, policy, recommendation, or guideline) for AI use or implementation in LMICs.
• Score 3: Papers proposing one or more solutions (e.g., strategy, framework, initiative, policy, or guideline) for AI use or implementation in LMICs, but restricted to any research context in health (tuberculosis, child and adolescent health, cancer, etc.).
• Score 4: Papers proposing one or more solutions (e.g., strategy, framework, initiative, policy, recommendation, or guideline) for AI use or implementation in LMICs, not restricted to any specific research context in health.

Disagreements on study selection and data extraction were solved by consensus and discussion with other reviewers when needed.

### Data charting process

Data from eligible studies were charted using a data abstraction template designed for this review. In this template, there was detailed information on the types of research, the research contexts related to the applications in health and applications of AI, the origin of the studies (classified by country and by income), and the origin of the data used in the studies. The template structure (Table 2) is intended for having an overview of the trends and information sets within the topic at hand, which is the development of AI in LMICs for healthcare.

**TABLE 2:** Description of data charting for individual sources of evidence.

Item	Description and options	Primary studies example	Secondary studies example
Title	Paper’s title	Deep Learning Assistance for Tuberculosis Diagnosis with Chest Radiography in Low-Resource Setting	Artificial Intelligence in Health Care Laying the Foundation for Responsible Sustainable and Inclusive Innovation in Low and Middle Income Countries
Authors	List the paper’s authors and affiliations	Nijiati et al. (36)	Alami et al. (8)
Year	Publication year obtained from metadata	2021	2020
Type of study	According to the aim of the paper, classification of the studies is according to: Experimental studies, systematic reviews, strategies or guidelines, opinion papers, case reports, study protocols, clinical practice guidelines, and qualitative research.	Experimental studies	Strategies or guidelines
Technical (AI) contribution	Essential contribution of the paper, can be classified according to: Data-based diagnosis, AI model for image-based diagnosis, AI model for data-based diagnosis, AI model for image-based mortality assessment, AI model for data-based mortality assessment, AI model for data-based treatment.	AI model for image-based diagnosis	AI for LMIC
Health topic	Purpose of the contribution to human health describe by topic and category; e.g., digital health, tuberculosis, child and adolescent health, cancer, and maternal and newborn health	Tuberculosis Communicable diseases	Digital health Health systems
Summary	Describe the abstract highlighting the components of interest for the research.	In a rural area of China, the authors have a dataset of X-ray images for the detection of tuberculosis. Using a DL model, the authors find an increase in detection accuracy when using the model to assist clinicians.	They propose a five-block guide for developing and implementing AI-based healthcare technologies for LMICs. They discuss the benefits, risks, and challenges of AI-based health, and from this, they draw guidance.
AI-driven health intervention	Select the type of intervention that is being used for the health topic among diagnosis, mortality risk assessment, treatment, clinical decision support, health policy	Diagnosis	Health policy
Study’s origin	Name of the country and income classification	China Upper-middle-income economies	Canadá High-income economies
Score	Assign a score according to what is found in the abstract	3	4
Notes	Important information that needs to be taken into account	It is simple experimentation	They mentioned strategies

### Data items

Data was extracted from information only available in the abstract. In addition, details were recorded on the template regarding the research type (e.g., experimental studies, systematic reviews, strategies or guidelines, opinion papers, case reports, study protocols, clinical practice guidelines, and qualitative research); research context on AI applications (e.g., Data-based diagnosis, AI model for image-based diagnosis, AI model for data-based diagnosis, AI model for image-based mortality assessment, AI model for data-based mortality assessment, AI model for data-based treatment, AI application for LMICs, AI model for clinical decision support, mHealth for LMICs); research context in health (e.g., digital health, tuberculosis, child and adolescent health, cancer, and maternal and newborn health, among others), AI-driven health interventions (20) (e.g., diagnosis, mortality risk assessment, treatment, clinical decision support), study’s origin (e.g., low-income economies, lower-middle-income economies, upper-middle-income economies, high-income economies), ethical aspects (i.e., it is mentioned or not), data set (i.e., there is, there is not, there is and it is from LMICs).

### Synthesis of results

According to the two groups of papers organized for this scoping review, there are two complementary methods of summarizing the information found. For the primary studies group, a trend analysis was performed where information on the most used AI technology and the health context that was the most approached is presented. For the secondary studies group, a trend analysis was also performed on some features extracted along with an in-deep review of the papers having strategies for a narrative summary of the gaps, challenges, solution frameworks, initiatives, and strategies implementable for AI.

## Results

### Selection of sources of evidence

**FIGURE 1:**
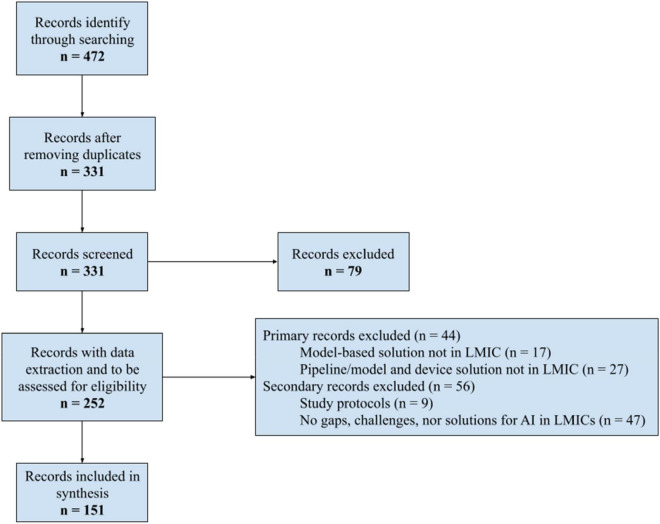
Flowchart of the study selection process.

After removing duplicates, 331 papers were screened by title and abstract. 79 articles were excluded based on this information, and data was extracted from the remaining 252 papers to assess their eligibility. From data extraction results, two groups of papers were identified from the type of study: one group with primary studies [see the effects in the variables of interest when introducing any intervention (28)] and the other group with opinion papers, systematic reviews, papers with strategies or guidelines, under the name of secondary studies. From the first group, 44 articles were excluded for the following reasons: 17 presented solutions based on models not addressed to LMICs, and 27 presented advanced solutions not directed to LMICs. From the second group, 56 papers were excluded: 9 were study protocols, and 47 papers did not report information on gaps, challenges, or solutions for AI implementation for healthcare in LMICs (Figure 1). Finally, the remaining 151 studies were considered eligible for this scoping review.

From now on, the description of the results is divided into the two papers groups found. The description of the primary studies group aims to describe the trends of the solutions that exist and involve the development of AI for health care and where research efforts are directed, especially in the context of LMICs. For the description of the papers belonging to the secondary studies group, a narrative summary of the findings regarding gaps and challenges for implementing AI in LMICs and their resulting solutions is provided.

### Characteristics of source evidence

#### Primary studies

In this first subsection, the trends in AI implementations in LMICs for healthcare are described, based on four aspects: the technological contribution, the data, the health context, and the interventions driven by AI for health.

**FIGURE 2:**
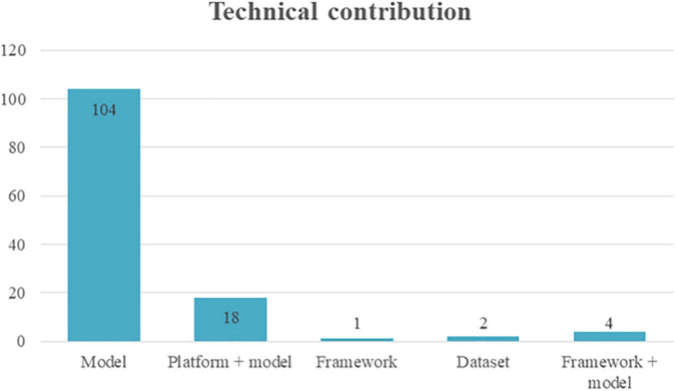
Distribution of the technical contributions.

**FIGURE 3:**
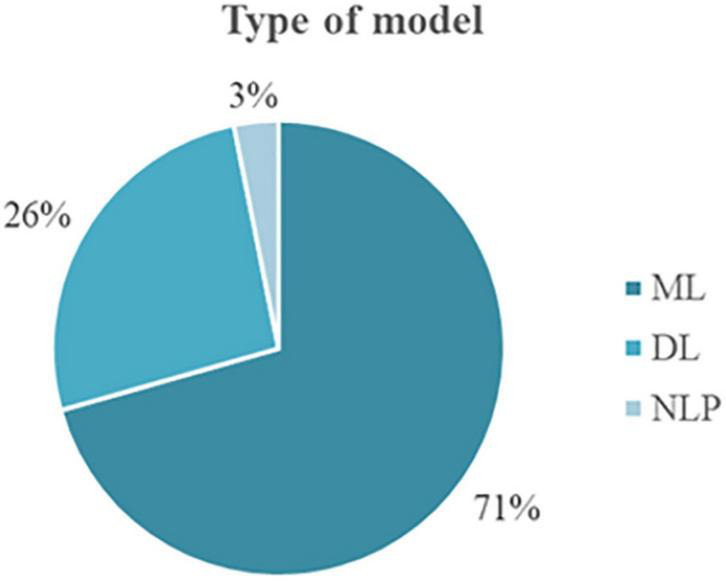
Distribution of the models.

As shown in Figure 2, from the 129 primary studies selected for this scoping review, 104 papers presented an AI model as the main technical contribution. Eighteen studies demonstrated an AI model plus its implementation in a technical platform (e.g., mobile, wearable platform). In four papers, the AI model was complemented by a framework, and the framework (without describing a model) was presented in only one contribution. Finally, the creation of a dataset was the contribution of two studies. From those studies presenting an AI model, 89 papers used machine learning (ML) models, 33 papers used deep learning (DL) models, and four papers used natural language processing (NLP) (Figure 3).

There are five types of platforms in the papers (Table 3). The most implemented are mobile applications in 10 papers, followed by wearables in four. Lastly, portable ultrasounds were developed in two papers, a web application and an enforcement system.

**TABLE 3:** Distribution of the platforms used.

Type of platform	
Mobile application	10
Wearable	4
Portable ultrasound	2
Web application	1
Enforcement system	1

Regarding the data used in the papers, two aspects were considered for the trend overview. The first aspect is the data type (Table 4). Clinical records data is the most used type, with 22% of the papers. Then, almost 40% of the documents used images, specifically radiology images (19% of the papers) and satellite images (7% of the papers). The remaining types of data are physiological signals (6% of the papers); demographic, biological, epidemiological, and laboratory data (each one, 5% of the papers); surveys (i.e., data that was acquired from large national or international efforts) with 4% of the papers; geographical data, text corpus, movement signals, and sounds (each one, 2% of the papers); and only one paper used videos as data source.

**TABLE 4:** Data found in the papers.

Type of data	
Clinical records data	28
Radiology images	24
Images	18
Satellite images	9
Physiological signals	8
Demographic data	7
Biological data	7
Epidemiological data	6
Laboratory data	6
Surveys	5
Geographical data	3
Text corpus	3
Movement signals	2
Sounds	2
Videos	1

**FIGURE 4:**
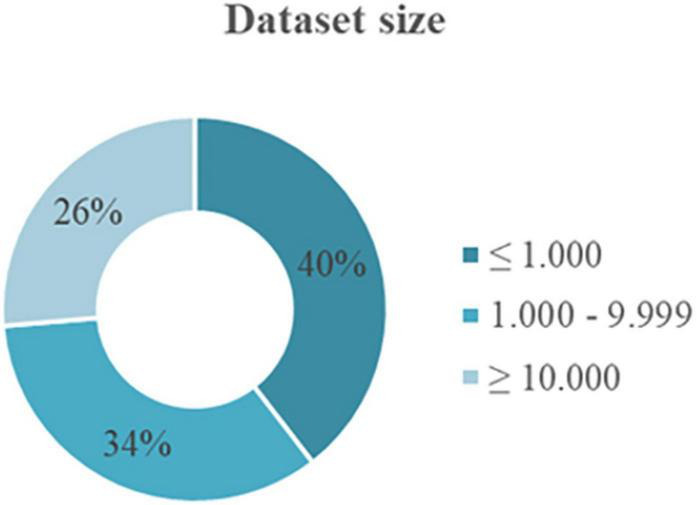
Distribution of the size of the datasets in the studies.

For the second aspect is the size of the dataset used in these primary studies. Figure 4 shows that most of the studies (51 papers) used small datasets, i.e., less than 1,000 instances. 44 papers deployed datasets that had between 1,000 and 9,999 instances, and 34 papers used large datasets with more than 10,000 instances.

The health topic addressed is the third aspect, when analyzing the trends in the studies found in this scoping review. In Table 5, there is the paper count for each health topic (31 topics) and each category of health topics (eight categories). For example, the most recurrent health topic is “maternal and newborn health,” with 16 papers approaching it. This topic corresponds to the most recurrent category, “life-course approach,” with 34 papers. The second and third places of the topics are “cancer” and “child and adolescent health,” respectively.

**TABLE 5:** Paper distribution according to health topic and category for primary studies.

Category	Health topic	Papers per topic	Papers per category
Life-course approach	Maternal and newborn health	16	34
	Child and adolescent health	13	
	Healthy aging	3	
	Disability and rehabilitation	2	
Non-communicable diseases	Cancer	15	31
	Mental health	5	
	Cardiovascular diseases	4	
	Diabetes	4	
	Chronic respiratory diseases	3	
Communicable diseases	Tuberculosis	12	20
	Vector-borne and parasitic diseases	3	
	HIV/AIDS	2	
	Others	2	
	Hepatitis	1	
Disease prevention	Violence and injuries	6	12
	Vaccines and immunization	3	
	Nutrition	1	
	Physical activity	1	
	Other	1	
Health systems	Digital health	6	10
	Blood safety	1	
	Health services delivery	1	
	Health technologies and medicines	1	
	Primary health care	1	
Environment and health	Urban health	4	10
	Transport and health	3	
	Climate change	1	
	Housing and health	1	
	Water and sanitation	1	
Health emergencies	COVID-19 outbreak	7	7
Health determinants	Social determinants	5	5

**FIGURE 5:**
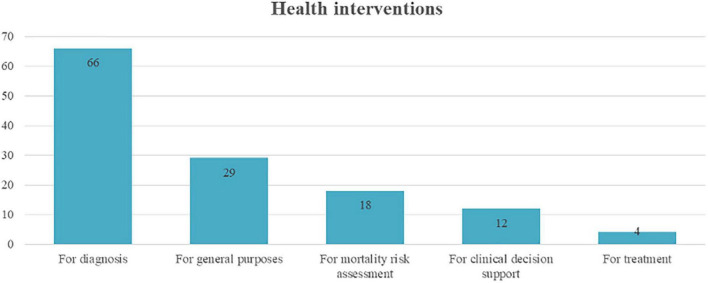
Papers using different health interventions.

Finally, the health interventions driven by AI mostly focused on diagnosing different diseases and conditions. In 66 papers, the authors addressed in their solution this intervention. Next, the AI solution considered in the paper was used for general purposes (29 papers), for example, age estimation and population density or distribution. The remaining interventions were mortality assessment with 18 papers, clinical decision support with 12 papers, and treatment with 4 papers (Figure 5).

**FIGURE 6:**
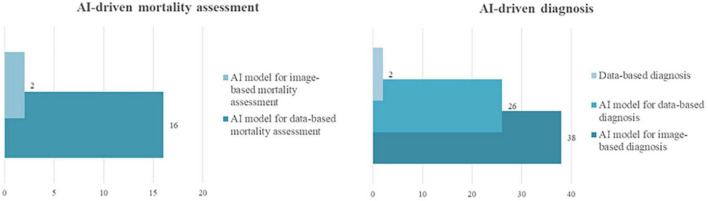
AI-driven approaches to health interventions.

From this data, different methods for diagnosis were implemented in the primary studies, precisely (i) the construction of datasets for diagnosis (i.e., data-based diagnosis) with two papers, (ii) the modeling of data (such as clinical or laboratory data) for diagnosis with 26 papers, and (iii) the modeling of images for diagnosis with 38 papers. Also, the information source for mortality assessment to build the model was based on images (2 papers) and clinical data (16 papers). Information on both approaches is presented in Figure 6.

Since the scope of the primary studies explored should be within the context of LMICs, results on the origin and affiliations of the authors of the papers were obtained as well.

**FIGURE 7:**
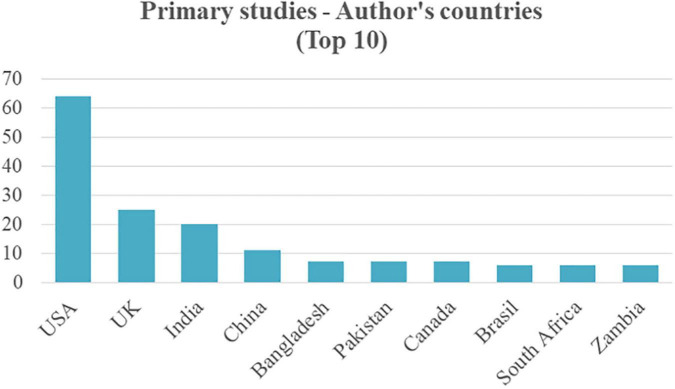
Top 10 of the author’s countries and affiliations.

Although the chosen papers use datasets from LMICs, the results show that many authors and affiliations belong to countries qualified as HICs. For example, in Figure 7, the USA leads the count with 64 papers that have authors affiliated with its universities and research centers, followed by the United Kingdom (UK) with 25 articles. Both countries are of course classified as HICs.

#### Secondary studies

This second subsection presents some highlights about the secondary studies group. First, Table 6 shows the distribution of the health topics; it has different results from the primary studies group. For example, the most common health topic was “Digital health”, defined by the WHO as an umbrella term encompassing e-health interventions for strengthening health systems toward universal healthcare coverage.

**TABLE 6:** Paper distribution according to health topic and category for secondary studies.

Category	Health topic	Papers per topic	Papers per category
Health systems	Digital health	14	15
	Health systems financing	1	
Environment and health	Social inequalities in environment and health	3	3
Communicable diseases	Vector-borne and parasitic diseases	1	2
	Others	1	
Health emergencies	COVID-19 outbreak	1	1
Non-communicable diseases	Mental health	1	1

**FIGURE 8:**
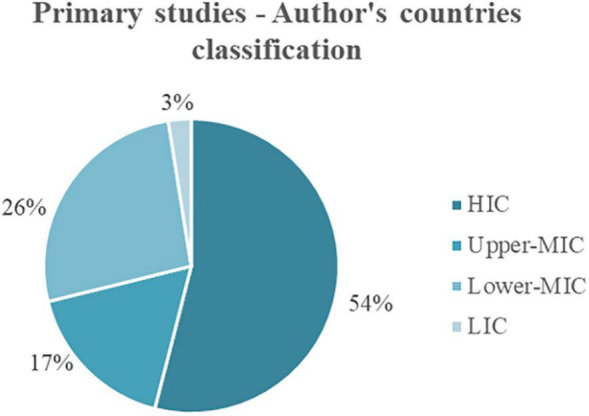
Distribution of author’s countries according to income classification.

From the list of countries obtained, 54% correspond to HICs, and only 3% correspond to Low-Income Countries (LICs). The remaining distribution is for Upper-Middle-Income Countries (Upper-MICs), with 17% of affiliations, and Lower-Middle-Income Countries (Lower-MICs), with 26% (Figure 8).

**FIGURE 9:**
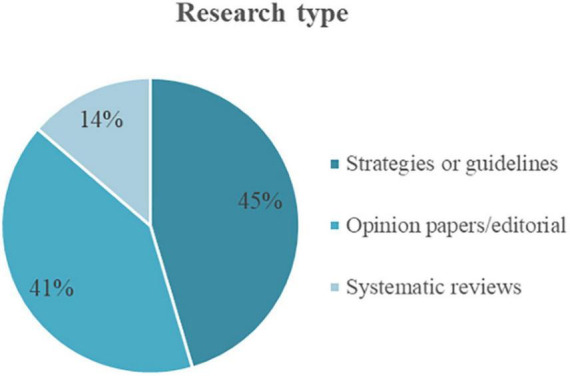
Distribution of the research type within the solution studies.

Figure 9 shows the type of research within this group of papers. Ten papers were found to have strategies or guidelines in the context of AI implementation policies in LMICs. Nine were opinion papers or editorial papers, and three were systematic reviews with important conclusions.

**FIGURE 10:**
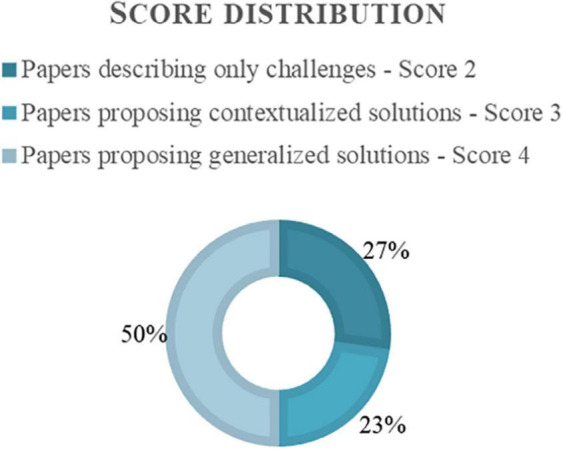
Distribution of the score within the solution studies.

Secondary studies established a score for eligibility (explained in the Methods section), as presented in Figure 10. Six papers were scored for only having challenges, five papers for presenting challenges and solutions for a specific problem or context, and 11 papers received a four-point score for proposing general solutions and contributing greatly to this scoping review.

**FIGURE 11:**
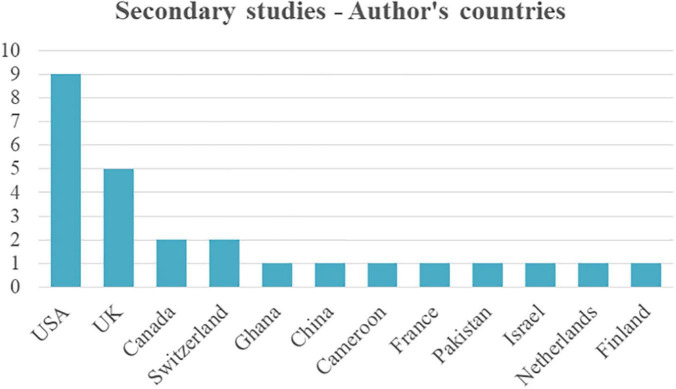
Top of the author’s countries and affiliations.

As with the primary studies, for the group of secondary studies, information on the countries and affiliations of the authors of the reviewed works were extracted, obtaining a similar result. In Figure 11, the complete list of countries is found, and again USA and UK lead the count.

**FIGURE 12:**
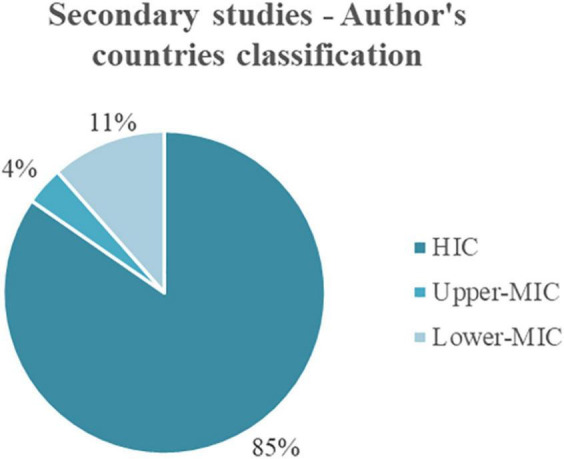
Distribution of author’s countries according to income classification.

Similarly, the countries that belong to the HICs classification present the majority of results, in this group, with 85% of the papers. The remaining is divided into Upper-MICs with 4% and Lower-MICs with 11% of the papers (Figure 12).

### Synthesis of individual results

For the description of the papers belonging to the secondary studies group, a narrative summary of the findings regarding gaps and challenges for implementing AI in LMICs and their resulting solutions is provided.

#### Dimensions and challenges

Forty different challenges were identified in the analyzed studies. They were grouped into eight categories: Data Quality, Context awareness; Regulation and Legal Frameworks; Education and Change Resistance; Financial Resources; Methodology; Infrastructure and connectivity; and Scalability. Each one of the challenges is detailed in Table 7.

**TABLE 7:** Set of challenges identified by domain with the respective description.

Challenges		Description
Data Quality	Accuracy	Accuracy means the degree to which data correctly represent a concept in a specific context (37). Therefore, AI algorithms should be trained and evaluated using local data.
	Consistency	Measures the coherence of data concerning the same or other data sources in a specific context of use (37). Accuracy, consistency, completeness, credibility, and currentness are other attributes that avoid deploying GIGO algorithms (Garbage in, Garbage out) in LMICs.
	Credibility	Determines how true and believable data is by users in a specific context of use (37). It is an essential attribute, especially in LMICs contexts, where many healthcare professionals are reluctant to use AI/ML technologies.
	Availability	is a measure of the capability of data to be retrieved by authorized users (humans or applications) in a specific period or context (37). The availability of quality data for training and evaluation is a frequently mentioned gap in LMICs.
	Diversity	Data diversity guarantees that data provides enough information to train AI/ML models. It maximizes the learning process, so ML models are fitted to the data. A common challenge mentioned in the literature data AI models are typically trained with HICs data, with different demographic characteristics, diseases, and contexts.
	Openness	This means that data is available to anyone for free, including permission for re-use and redistribution (38).
Context-awareness	Contextual applicability	AI models and solutions must be validated before deployment using data acquired from the local context in LMICs.
	Diseases priorities	AI interventions should be planned to consider the burden of disease in the local context.
	Appropriateness	It is the matching between a machine learning model and the target population. It encompasses deciding the application scenarios, policies, and liability, among others, in the local context (22).
	Bias (demographic, economic, racial)	It is defined as a systematic error that causes to favor one outcome over another. In terms of algorithms, bias is caused by an undesired dependence on a specific data attribute in the data, e.g., gender, race, or religion (22). In addition, (15) introduces the concept of contextual bias, in which the systematic error is caused by AI algorithms created in HICs and deployed in LMICs where the health contexts are different, e.g., differences in health risks, treatment, demographics, economy, etc.
	Fairness	Unlike bias, a mathematical construct, fairness is a socially defined concept in which the impact of an AI model in decision-making processes is assessed against a set of legal or ethical principles, which are often contextually dependent, e.g., it depends on the local government and culture.
	Generalizability	Defined as an attribute of ML models to determine how well it’s trained to classify or predict new data correctly. Generalizability is relevant in LMIC due to data, infrastructure, and knowledge limitations to build new local models.
	Explainability	It is the level of understanding of how the system produced a specific result (39).
Education and change resistance	Training different stakeholders	The lack of training and understanding of AI technologies by different stakeholders (decision-makers, developers, health professionals, citizens, patients, communities, etc.) is a limitation in LMICs. Different stakeholders are involved in AI policies, regulations, research, design, implementation, and deployment.
	Insufficient motivation	Healthcare professionals and patients know that AI technologies have surpassed the human capacity to accomplish some administrative and clinical workflows. However, there is still a lack of motivation to use these tools, especially because of the unsolved ethical and regulatory concerns and the perceived risks of using AI applications in healthcare. Also, healthcare staff and front-line workers in LMICs still do not benefit from collecting and aggregating more and more data.
	Change resistance	Despite the inevitable advent of AI technologies, there is still a fear that AI will replace the work of healthcare professionals and staff.
Methodology	Reporting and methodological standards	Reporting and methodological standards are required for AI health interventions in LMICs. It includes standardized methods and indicators to evaluate the added value of AI interventions over current standards of care.
	Human-centered design	Human-centered design (HCD) is an approach to designing and developing interactive products, services, and experiences, driven by the user. AI systems need to involve different stakeholders to guarantee success. However, HCD is a practice that is not frequently used in AI solutions.
	Certification	Regulation and certification processes are necessary to promote the advance and large-scale deployment of AI/ML technologies. Also, to guarantee patient safety and effectiveness.
Regulation and legal frameworks	Informed consent	A typical healthcare scenario means an agreement between a patient and the healthcare provider about a medical condition and the options for treatment. Informed consent when AI/ML technologies are used in healthcare scenarios implies that patients are informed about this fact.
	Privacy	In the context of health, data management is defined as the right of a person to maintain their private life, avoiding any illegal gathering and use of their data (40). Therefore, AI/ML algorithms and solutions should respect health data privacy, supported by proper patient consent.
	Ethical issues	An ethical issue is a behavior that is not in accordance with accepted principles of right or good conduct in data management and the decision-making process in healthcare. ML policies and legal frameworks should protect individuals against unethical behaviors.
	Data security	Information security protects against unauthorized access, use, disclosure, modification, or destruction of information (40). Therefore, from a technical perspective, AI/ML-based solutions or eHealth infrastructures should protect users against these problems.
	Liability	It is a current obligation acquired by an organization due to events that occurred in the past (41). Liability is a challenge for healthcare organizations, especially healthcare providers using AI-based solutions, because of the unexplainably of algorithms (black-box algorithms) and lack of unclear policy and legal frameworks in LMICs.
	Under-representation	Data on certain patient groups in LMIC are frequently not present in local databases due to the inequalities in providing health care services or health insurance coverage. Therefore, the above contribute to systematic biases causing non-representative conclusions (12).
	Confidentiality	It is a guarantee that data is not made available or disclosed to unauthorized actors (42).
	Trust	In the interaction between two actors, one actor assumes that the other actor will behave exactly as the first actor expects (42). A challenge in LMICs is the fear of patients’ and practitioners’ trust in the decisions or advice made by AI/ML systems.
	Accountability	The accountability of an AI/ML system is the guarantee that the actions performed by that system are traceable (43). Therefore, accountability is critical in “black box” ML models.
	Equity	Low socioeconomic populations have less access to healthcare. Therefore, databases and registries have less data on these minority populations, causing inequalities.
	Governance	Data governance requires a systematic process to guarantee data quality (Consistency, Credibility, Availability, Diversity, and Openness). Policies and processes for data governance and data ownership are limited in LMICs.
	De-identification	De-identification is the process of removing identifying data of a subject, eliminating the possibility of recognizing that subject in a specific context (44). De-identification is a challenge because many LMICs have different definitions of personal information. Moreover, when common characteristics aggregate de-identified data, there is a risk of identifying personal information (45).
Financial resources	Health system priorities	Decisions on allocating resources for Digital Health programs, particularly AI technology, are not frequently made, prioritizing local needs and the burden of diseases. However, the decisions in LMICs are sometimes made considering data availability and donors’ funding priorities.
	Sustained funding	A lack of sustained funding restricts the development and adopting of digital health technology LMICs.
Infrastructure and connectivity	Poor connectivity	Despite big advances in connectivity, especially mobile networks, there are still many rural areas in LMICs where connectivity is an issue. Especially broadband connections.
	Electronic Health Record Systems/ Patient Registers	Integrated Electronic Health Record systems (EHR) are a challenge that still needs to be solved, especially in rural areas and countries where the health system is fragmented. Also, secure access to EHR data is problematic because of the lack of interoperability infrastructures and data-sharing policies. Patient registries are generally more complete than EHR systems, but due to their complexity for data processing, needed infrastructure and maintenance cost are scarcer in LMICs.
	Computer capacity	Increased computer and storage capability is one factor that has boosted AI solutions. However, access to high-performance infrastructures is costly, especially for LMICs, where providers are typically unavailable in the country.
	Interoperability–Data aggregation	International standards for interoperability and controlled vocabularies and terminologies are in place. However, the implementation of interoperability solutions in LMICs, connected to the implementation of robust integrated EHR systems, lags behind current developments in HICs.
Scalability	Scalable solutions	Scalable AI/ML solutions can increase their functionalities, responding to contextual demands. Therefore, scalable solutions are essential for widespread health intervention and support dynamic and diverse health contexts in LMICs.
	Cost-effectiveness	Digital health interventions, particularly AI-based interventions, must demonstrate cost-effectiveness, especially in LMICs where resources are scarce.
	Continuous impact evaluation	Health outcomes of AI interventions have to be continuously measured. This is complex and costly, especially in LMICs, considering the deficient infrastructures, digitalization, research agendas, and development environments.

#### Solutions to challenges

**FIGURE 13:**
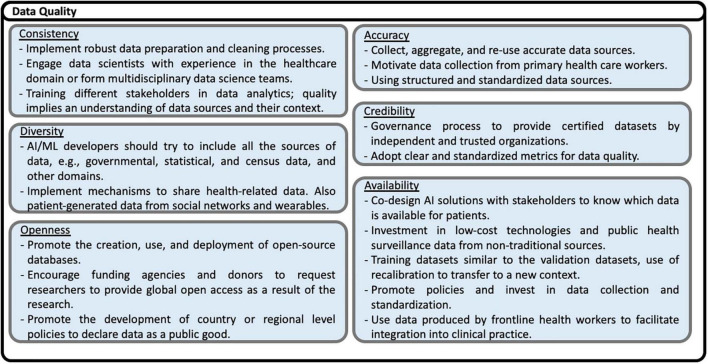
Solutions to challenges for Data Quality.

**FIGURE 14:**
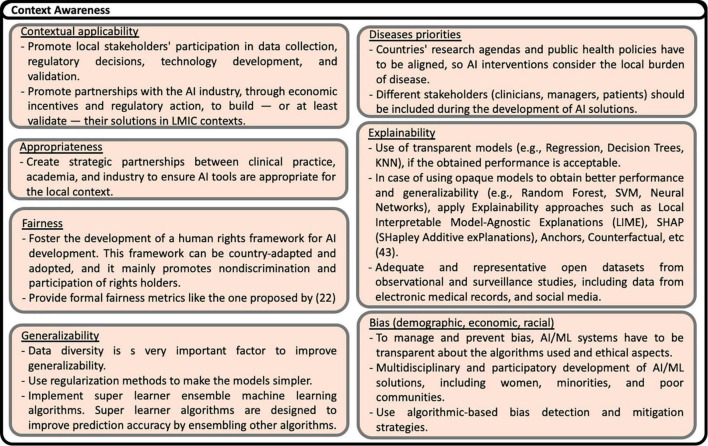
Solutions to Context-awareness challenges (22, 43).

**FIGURE 15:**
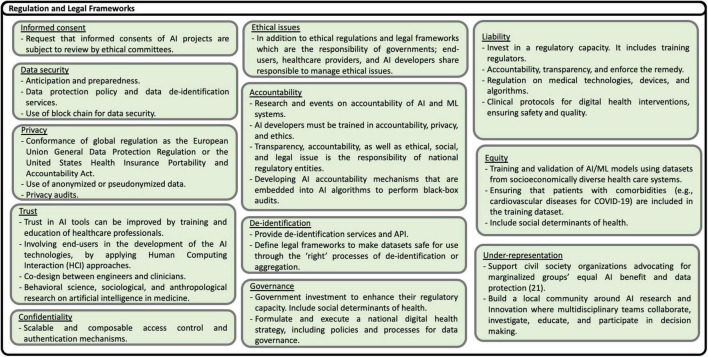
Solutions to Regulation and Legal Frameworks challenges (21).

**FIGURE 16:**
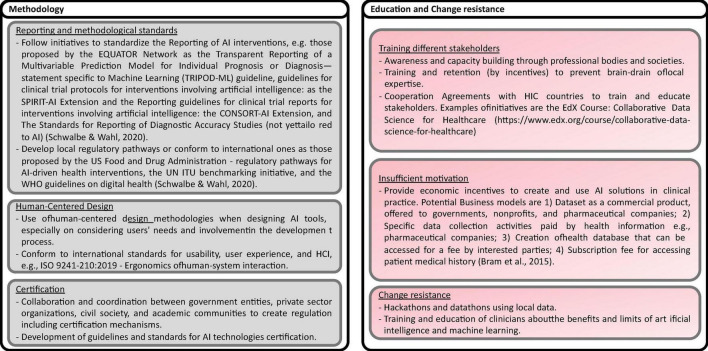
Solutions to Methodological and Education challenges (18, 20).

**FIGURE 17:**
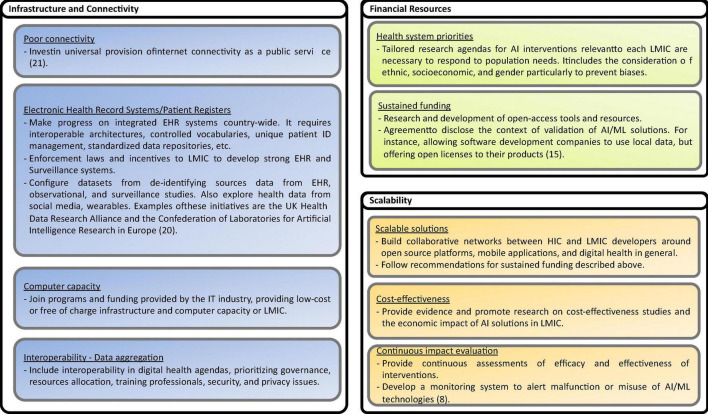
Solutions to Infrastructure and connectivity, Financial Resourced, and Scalability challenges (8, 15, 20, 21).

Based on the recommendations presented in the studies analyzed, including some reflections of the authors of this scoping review, eighty nine possible solutions to the challenges are identified. The solutions are shown in Figures 13–17, according to each of the eight dimensions.

## Discussion

### Summary of evidence

#### Primary studies

• Technical contribution

The standing-out contribution is Al models. The solutions are still incipient since it is not yet possible to determine if the models can be generalized regardless of the context. The models should be generalizable from the data science and artificial intelligence perspectives; nevertheless, there are problems, such as model discrimination for special or unprecedented cases. Eventually, implementing the models developed around healthcare can help streamline clinical care by properly monitoring the regular process of a defined treatment. However, human medical intervention remains essential for cases where the model cannot accurately classify or predict.

Given that implementation of good models is still in experimentation, it is clear why ML models, whether for data or images, are the most used. On the other hand, DL-based models are less common because two primary features are needed to develop them: having a large image bank, for example, those used in diagnosis (X-rays), and having a high computational capacity since the implementation of neural networks demands a lot of resources from the device or the cloud that runs the model.

Regarding the generation of platforms and models, the second contribution by the number of papers, it is noteworthy that mobile devices’ solutions are widely implemented in LMICs contexts. In such contexts, the device penetration is high where the access to healthcare in different facilities is shallow or none (29).

• Data

Clinical data and radiology images are the most commonly used data types in experimental studies, which makes sense from a clinical and healthcare point of view: these data are the closest to representing medical knowledge for the diagnosis and consequent treatment giving certain conditions in a patient’s health.

It is interesting to note that images are one type of data. Most of these correspond to photos taken with mobile devices, consistent with developing models and platforms based on this technology. On the other hand, physiological signals can be considered real-time indicators of a person’s current state and help avoid bias or subjectivity in the information provided by a patient. Although they have this advantage, their processing is complex and depends on the type of device to collect the signal.

The type of data from surveys draws attention to the effort needed to obtain such data, both nationally and internationally, since collection requires logistical efforts and high economic capacity.

The distribution in the data quantity used in the selected experimental studies makes sense since verification of a model function is necessary to have contextualized data. This is an extra effort for the researchers; it is not easy to carry out in specific LMICs contexts in many cases. Those papers using large amounts of data do so because of their availability and not having to perform the collection work.

• Health context

The category with the most references is “life-course approach,” which includes “maternal and newborn health” and “child and adolescent health.” In the context of LMICs, women’s pregnancy suffers from inequalities in care, especially in rural and marginalized areas. The physical and psychological effects of this lack of care lead to the deterioration of the newborn’s and her mother’s health. In addition, in LMICs, there are high rates of child malnutrition, which has, consequently, effects on the health of children and adolescents and, therefore, uncertainty in the future development of these countries.

The following categories correspond to non-communicable and communicable diseases. These groups include chronic diseases such as cardiovascular diseases, cancer, chronic respiratory disorders, and diabetes. These diseases are highly addressed in experimental studies and are according to the data available from organizations such as the WHO about their prevalence in LMICs.

Cancer, for example, with a high prevalence in LMICs, presents many developments and implementations as researchers seek tools to generate an early diagnosis of the disease and, therefore, a greater probability of treatment success. Developments to preserve people’s mental health are also highlighted, especially with the global context of COVID-19 and the isolation measures taken to counteract the contagion’s negative effects. These measures have a huge impact on mental health (30).

Although the incidence of tuberculosis has been falling in recent years, it is still one of the leading causes of death globally (31). As a result, many efforts around early detection are being made to decrease its prevalence, especially in LMICs contexts, where funding for detection and treatment is far below what is needed (32).

• AI-driven health interventions

Experimental studies’ primary purpose is to diagnose diseases, especially early and accurate diagnosis. Another purpose is the mortality assessment, mostly to avoid newborn deaths, which has a high rate in LMICs (33). Finally, clinical decision support systems are an important target for implementation; developing these systems can reduce hospitalization times, optimize treatment, and reduce work stress for health professionals (34).

• Country of affiliation

When extracting the affiliation data of the authors of experimental studies and contrasting them against the country’s classification to which the research and development institutions belong, it was interesting to see that the developments of experimental studies are conceived mostly from HIC. However, the datasets were collected in LMICs contexts. A researcher’s purpose is to impact the environment by detecting problems and proposing solutions driven by the characteristics of the context. Economic resources are decisive in the construction of solutions. For this reason, establishing relations between institutions is convenient to equate global efforts in this type of research to eradicate different personal and public health conditions.

#### Secondary studies

• Data Quality challenges

Data quality encompasses many aspects of data, from intrinsic to extrinsic. Accuracy is the correct representation of the health-related concepts considering the local LMICs context. Therefore, AI algorithms should be trained and evaluated using local data. Electronic Health Records (EHR) data and data registries are the preferred data source. Also, collecting data from primary healthcare workers improves the quality of data sources. Low-cost technologies such as sensors, phone applications, and public health surveillance data from non-traditional sources also improve data availability and diversity. Consistency, completeness, credibility, and currentness are other attributes that avoid the deployment of Garbage in, Garbage out (GIGO) algorithms. Maintaining quality data implies implementing robust data preparation to manage and prevent bias and cleaning processes engaging data scientists and multidisciplinary teams with knowledge and experience in the healthcare domain. Training different stakeholders, another domain explained below, is also important to improve data quality because it implies understanding the data sources and their context. The governance process includes data quality policies to provide certified datasets by independent and trusted local and international organizations. Quality improvement implies using clear and standardized metrics for data quality as proposed by several international standards and initiatives in software engineering. Co-design AI solutions with users, physicians, patients, and clinical managers contribute to improving data quality. Recommendations include implementing mechanisms to share health-related data and promoting the creation, use, and deployment of open-source databases such as MIMIC-III, a critical care database.

• Context-awareness challenges

Contextual awareness means that AI models and solutions must be validated using data from the local context in LMICs. A common gap mentioned in the literature is that most AI models used in LMICs are typically trained with HICs data with different demographic characteristics and contexts. Context awareness also implies an appropriate emphasis on application scenarios, policies, and disease priorities to prevent bias and promote model generalizability and explainability. Actions addressing contextual awareness challenges include local stakeholders’ participation in data collection, regulatory decisions, technology development, and validation. Creating strategic partnerships between clinical practice, academia, and industry is foremost important. Also, AI Interventions should be planned to consider the burden of disease in the local context. To manage and prevent bias, AI/ML systems must be transparent about the algorithms used and ethical aspects of managing and preventing bias. In this direction, to favor explainability, transparent models are preferred if the obtained performance is acceptable. In the case of using black-box models, it is suggested deploying explainability approaches such as Local Interpretable Model-Agnostic Explanations (LIME), SHAP (SHapley Additive exPlanations), Anchors, Counterfactual methods, among others. Data diversity is a very important factor in improving generalizability. ML techniques, such as regularization methods, make the models simpler.

• Challenges in the regulation and the provision of legal frameworks

Local regulation and legal frameworks, strategies, and policies are fundamental to successfully deploying AI/ML solutions. The regulation includes the provision of privacy, security, informed consent, ethics, liability, confidentiality, trust, equity, and accountability policies. In addition, local governance and leadership are necessary to promote and execute national AI strategies included within digital health strategies at country, regional, and local levels. Recommendations to overcome security, privacy, safety, trust, and ethical issues include making mandatory before funding any intervention, the approval by ethical committees of informed consent, and clinical protocols.

Also, conformance of local policies to international regulation, scalable and composable access control and authentication mechanisms, anonymized or pseudonymized data, and mandatory privacy audits are necessary. ML policies and legal frameworks should protect individuals against unethical behaviors. In addition to ethical regulations and legal frameworks, which are the responsibility of governments, end-users, healthcare providers, and AI developers, share responsibility for managing ethics. Liability is a challenge for healthcare organizations, especially healthcare providers using AI-based solutions. Therefore, explainable ML models have to be provided. For example, data of certain patient groups in LMICs are frequently not present in local databases, caused by existing inequalities in the provision of health care services and low health insurance coverage. Inequality is also present when AI interventions take care mainly of the diagnosis but not the treatment and follow-up of patients. Trust in AI tools can be improved by training and educating healthcare professionals and involving end-users in developing AI technologies applying Human-Computing Interaction (HCI) approaches. Moreover, AI developers must be trained in accountability, privacy, and ethics.

• Education and change resistance challenge

Limitations in training and education of different stakeholders (decision-makers, developers, health professionals, citizens, patients, and communities) prevent the understanding, use, policy-making, research, and innovation of AI technologies in LMICs. Potential solutions include capacity building through professional bodies and societies, training and retention to prevent brain-drain of local expertise, and cooperation agreements with HICs to train and educate stakeholders. In addition, insufficient motivation to use AI/ML tools is a major concern, especially because of the unsolved ethical and regulatory concerns and the perceived risks of using AI applications in healthcare. One alternative is providing economic incentives to create and use AI solutions in clinical practice. Also, the innovative implementation of business models around data collection and aggregation, which, ethically managed, could be an alternative incentive for building AI solutions. Another critical aspect is change resistance, mainly due to the fear that AI will replace the work of healthcare professionals and staff. Training and education of clinicians about the benefits and limits of artificial intelligence and machine learning, and more recently, hackathons and datathons events using local data, have been demonstrated to be effective actions.

• Methodological challenges

Solutions to methodological challenges covered reporting and methodological standards, the Human-Centered Design (HCD) of solutions, and the adoption of certification mechanisms. Reporting and methodological standards are required for AI health interventions in LMICs to evaluate AI interventions’ impact and added value over current standards of care. Several initiatives are being developed, becoming the standard de fact approaches for reporting AI interventions. One example is the EQUATOR Network, which has proposed guidelines for reporting interventions involving artificial intelligence. In the same direction, the United Nations (UN), the International Telecommunication Union (ITU), and the WHO are proposing guidelines on digital health interventions involving AI technologies. To prevent bias and guarantee accuracy, diversity, and trust, AI systems need to be contextually aware and involve different stakeholders in all stages of development. Methodologies to support these challenges are HCD approaches. Multidisciplinary work requires collaboration and coordination between government entities, private sector organizations, civil society, and academic communities. Furthermore, certification processes are necessary to promote the advance and large-scale deployment of AI/ML technologies. Also, to guarantee patient safety and effectiveness.

• Data infrastructure and connectivity challenges

The increased use of mobile networks has improved connectivity in LMICs. However, many rural areas in LMICs countries lack continuous Internet access. Investment in the universal provision of internet connectivity is a priority. Regarding data infrastructures, the availability of electronic health records and secure access to EHR data is still an unsolved problem in many countries and regions. Therefore, governments, healthcare providers, professional associations, and other actors should promote the construction of national eHealth infrastructures, including interoperability platforms, the adoption of international vocabularies, terminologies, and ontologies, and the implementation of unique patient ID management systems and standardized data repositories. In countries where infrastructure and connectivity do not progress as desired, enforcement laws, on the one hand, but incentives to develop strong EHR and surveillance systems are possible alternatives. On the other hand, the demand for computing capacity and storage capability increases. Join programs and funding provided by the IT industry, providing low-cost or free-of-charge infrastructure and computer capacity to LMICs is a viable alternative.

• Financial Resources allocation challenges

The allocation of adequate and sustained financial resources is one of the challenges frequently mentioned in LMICs for implementing digital technologies in general. In many LMICs, Digital health and AI/ML technologies are not a priority, or the decision on the allocation of scarce resources is not frequently made, prioritizing local needs and the burden of diseases but considering data availability and donors’ funding priorities. Potential solutions to overcome these challenges are establishing national research and innovation agendas for AI interventions responding to population needs. It includes the consideration of ethnicity, socioeconomic, and gender, particularly to prevent biases. In addition, research and development of open-access tools and resources could foster AI interventions’ experimentation, mainstreaming, and scale-up. One alternative in the agreement with international and local software development enterprises is to offer open licensing and free training of their products.

• Scalability challenges

Scalable solutions are important for extending AI-based health interventions and supporting the dynamic and diverse health contexts in LMICs. To be scalable, AI-based interventions demonstrate cost-effectiveness, health system efficacy, and economic impacts. Building collaborative networks between HICs and LMICs developers around open-source platforms, mobile applications, and digital health is promising. Health outcomes of AI interventions have to be continuously measured. This is complex and costly, especially in LMICs, considering the inadequate infrastructures, digitalization, research agendas, and development environments. Another strategy identified is to develop monitoring systems to report malfunction or misuse of AI/ML technologies.

The above recommendations provide a framework to be considered by health IT project at different levels, from pilot to national health information systems. However, the selection of the most relevant challenges depends on the maturity level of each project and especially on the context of the use of digital health solutions.

### Limitations

One of the strengths of this scoping review is the strict adherence to the recommendations provided by the PRISMA extension for scoping reviews. In addition, PRISMA provides detailed descriptions for conducting scoping reviews systematically, allowing readers to assess the adequacy of the sources used, thus ensuring the reliability of the findings. It also allows for repeatability and updating of the review.

This scoping review has some limitations. First, reviewing the papers was made between two reviewers; even though this guarantees a less biased process, disagreements were solved between the reviewers and not by a third party. Second, the reviewing process took longer than expected due to the data extraction and charting. It probably could lead to outdated source data; this scoping review was a big undertaking, and results are only up to date as of December 2021. However, we presume that the results are not likely to be outdated soon if the tendency of LMICs researchers to produce just a few studies of AI use and application in healthcare does not change. Third, the scientific quality of the studies included in the review was not assessed. Many studies included have design limitations. However, including, for example, only randomized controlled trials would have extremely limited the number of studies to analyze.

## Conclusion

This scoping and narrative review systematically characterized current AI healthcare implementations in LMICs, describing their technological contribution, data used, health context, and type of health interventions. It was found that most studies proposed experimental machine learning models followed by Deep Learning based models. However, few studies deployed the models, and those deploying them implemented them mainly on mobile platforms. Regarding data sources characterization, clinical records data and radiology images are the most commonly used data types in experimental studies. Most images correspond to photos taken with mobile devices. Most datasets are small due to the high cost of collecting local data. Bigger datasets correspond to international projects or organizations collecting data in LMICs or using open data such as satellite images or surveys.

Regarding the health context of AI applications, most interventions addressed maternal, newborn, and child and adolescent health. The second most common interventions were cancer, mental health, and cardiovascular diseases in the group of non-communicable diseases. Finally, tuberculosis, COVID-19, vector-borne and parasitic diseases, and HIV; accounted for the group of infectious diseases. Studies addressing violence and injuries were also prevalent. Regarding the type of intervention, the primary purpose of the experimental studies was the diagnosis of diseases, followed by mortality assessment.

This review study adds to the current literature a detailed description of gaps, challenges, and possible solutions for AI deployment in the healthcare systems of LMICs. Research on applying AI and ML to healthcare interventions in LMICs is growing; however, apart from very well-described ML methods and algorithms, several issues need to be addressed to scale and mainstream experimental and pilot studies. Those challenges include improving the quality of existing data sources, training and modeling AI solutions based on contextual data; and implementing privacy, security, informed consent, ethics, liability, confidentiality, trust, equity, and accountability policies. Also, potentiating widespread AI solutions in LMICs requires a robust environment with trained stakeholders, methodological standards for data creation, research results reporting, and product certification.

A very important lesson learned regarding the design and management in translational health ecosystems is the need to advance from a data focus to a concept and knowledge focus, i.e., replacing data sharing by knowledge sharing. This holds for all aspects and sections presented in this paper. In that context, we refer once more to the introductory paper of this Special Issue (1) or to (35).

## Data availability statement

The data sources used in this study are available in Scopus and Web of Science Databases. Further inquiries can be directed to the corresponding author.

## Author contributions

DL and CR-O conceived and designed the study, collected data, did the data analysis, and drafted the manuscript. BB addressed the general challenges LMICs face in their IT strategies for health. All authors revised, reviewed, and approved the manuscript.
